# The complete mitochondrial genome sequence of *Oryctes rhinoceros* (Coleoptera: Scarabaeidae) based on long-read nanopore sequencing

**DOI:** 10.7717/peerj.10552

**Published:** 2021-01-13

**Authors:** Igor Filipović, James P. Hereward, Gordana Rašić, Gregor J. Devine, Michael J. Furlong, Kayvan Etebari

**Affiliations:** 1 School of Biological Sciences, The University of Queensland, St. Lucia, Australia; 2 Mosquito Control Laboratory, QIMR Berghofer Medical Research Institute, Brisbane, QLD, Australia

**Keywords:** Mitochondrial genome, Scarabaeidae, *Oryctes rhinoceros*, Coconut palm pest, ONT MinION, Biosecurity and pest management

## Abstract

**Background:**

The coconut rhinoceros beetle (CRB, *Oryctes rhinoceros*) is a severe and invasive pest of coconut and other palms throughout Asia and the Pacific. The biocontrol agent, *Oryctes rhinoceros nudivirus* (OrNV), has successfully suppressed *O. rhinoceros* populations for decades but new CRB invasions started appearing after 2007. A single-SNP variant within the mitochondrial *cox1* gene is used to distinguish the recently-invading CRB-G lineage from other haplotypes, but the lack of mitogenome sequence for this species hinders further development of a molecular toolset for biosecurity and management programmes against CRB. Here we report the complete circular sequence and annotation for CRB mitogenome, generated to support such efforts.

**Methods:**

Sequencing data were generated using long-read Nanopore technology from genomic DNA isolated from a CRB-G female. The mitogenome was assembled with Flye v.2.5, using the short-read Illumina sequences to remove homopolymers with Pilon, and annotated with MITOS. Independently-generated transcriptome data were used to assess the *O. rhinoceros* mitogenome annotation and transcription. The aligned sequences of 13 protein-coding genes (PCGs) (with degenerate third codon position) from *O. rhinoceros*, 13 other Scarabaeidae taxa and two outgroup taxa were used for the phylogenetic reconstruction with the Maximum likelihood (ML) approach in IQ-TREE and Bayesian (BI) approach in MrBayes.

**Results:**

The complete circular mitogenome of *O. rhinoceros* is 20,898 bp in length, with a gene content canonical for insects (13 PCGs, two rRNA genes, and 22 tRNA genes), as well as one structural variation (rearrangement of *trnQ* and *trnI*) and a long control region (6,204 bp). Transcription was detected across all 37 genes, and interestingly, within three domains in the control region. ML and BI phylogenies had the same topology, correctly grouping *O. rhinoceros* with one other Dynastinae taxon, and recovering the previously reported relationship among lineages in the Scarabaeidae. In silico PCR-RFLP analysis recovered the correct fragment set that is diagnostic for the CRB-G haplogroup. These results validate the high-quality of the *O. rhinoceros* mitogenome sequence and annotation.

## Introduction

*Oryctes rhinoceros* (L.) (Coleoptera: Scarabaeidae: Dynastinae), also known as the coconut rhinoceros beetle (CRB), is an important agricultural pest causing significant economic damage to coconut and other palms across Asia and South Pacific. During the 20th century, human-mediated dispersal resulted in the distribution of *O. rhinoceros* expanding from its native range (between Pakistan and the Philippines) throughout Oceania (Catley, 1969). After the discovery and introduction of the viral biocontrol agent *Oryctes rhinoceros nudivirus* (OrNV) in the 1960s, most of the CRB populations in the Pacific islands have been persistently suppressed (Huger, 2005). However, after a biocontrol campaign failed to eradicate a newly established population in Guam in 2007, new *O. rhinoceros* invasions were recorded in Papua New Guinea (2009), Hawaii (2013) and Solomon Islands (2015) and more recently in New Caledonia and Vanuatu (Etebari et al., 2020). Worryingly, the new invasive populations have also been difficult to control by known OrNV isolates (Marshall et al., 2017), emphasizing the importance of actively overseeing and adapting the management programmes for this important insect pest.

The expansion pathways, dynamics and hybridization of invasive insect pests and other arthropods are commonly traced through the analyses of mitochondrial sequence variation (Wang et al., 2017; Rubinoff et al., 2010; Moore et al., 2013). In the absence of a mitogenome sequence, the universal barcoding region is often amplified with degenerate primers to investigate partial sequences of *cox1* and a limited number of other mitochondrial genes in the target species. However, analyses of such partial sequence data can fail to distinguish true mitochondrial lineages unless a sufficient number of genetic markers can be retrieved. Variation from a partial sequence of one mitochondrial gene (*cox1*) and one nuclear gene (*cad*) was not sufficient to allow confident hypotheses testing around *O. rhinoceros* invasion pathways (Reil, Jose & Rubinoff, 2016), but a single diagnostic SNP within the partial *cox1* gene amplicon has been used to distinguish the CRB-G haplotype from other haplotype that originally invaded the Pacific islands in the early 1900s (Marshall et al., 2017; Etebari et al., 2020). Here we report the first and complete mitogenome sequence assembly of *O. rhinoceros*, a genomic resource that will support the development of a comprehensive molecular marker toolset to help advance the biosecurity and management efforts against this resurgent pest.

The complete *O. rhinoceros* mitogenome assembly was generated using long-read Oxford Nanopore Technologies (ONT) sequencing and complemented with the short-read Illumina sequencing. The approach recovered all 37 genes (Cameron, 2014b) and a long non-coding (control) region (6204 bp) that was not recovered in a short-read (Illumina-based) assembly, likely because it contains different putative tandem repeats. Three domains with detectable transcription within the control region and the rearrangement of two tRNA genes (*trnI and trnQ*) were also identified. The high quality of the assembly was validated through the correct placement of *O. rhinoceros* within the Scarabaeidae phylogeny, transcription patterns from an independently-generated transcriptome dataset, and in silico recovery of a recently reported diagnostic PCR-RFLP marker. This is the first complete mitogenome for the genus *Oryctes* and the subfamily Dynastinae, and among only a few for the entire scarab beetle family (Scarabaeidae).

## Materials and Methods

### Sample collection, DNA extraction and ONT sequencing

An adult female *O. rhinoceros* was collected from a pheromone trap (Oryctalure, P046-Lure, ChemTica Internacional, S. A., Heredia Costa Rica) on Guadalcanal, Solomon Islands in January 2019 and preserved in 95% ethanol. Mrs Helen Tsatsia (Director of Research) and members of the research team at the Ministry of Agriculture and Livestock, Honiara, Solomon Islands Government facilitated the insect collection in Solomon Islands. Initially, the mitochondrial haplotype of the specimen was determined as CRB-G (Marshall et al., 2017) via Sanger sequencing of the partial *cox1* gene sequence that was amplified using the universal barcode primers LCO1490 and HCO2198 (Folmer et al., 1994). High-molecular weight DNA was extracted using a customized magnetic (SPRI) bead-based protocol. Specifically, smaller pieces of tissue from four legs and thorax (50 mm^3^) were each incubated in a 1.7 ml eppendorf tube with 360 μL ATL buffer, 40 μL of proteinase K (Qiagen Blood and Tissue DNA extraction kit) for 3 h at RT, while rotating end-over-end at 1 rpm. A total of 400 μL of AL buffer was added and the reaction was incubated for 10 min, followed by adding 8 μL of RNase A and incubation for 5 minutes. Tissue debris was spun down quickly (1 min at 16,000 rcf) and 600 μL of homogenate was transferred to a fresh tube, where SPRI bead solution was added in 1:1 ratio and incubated for 30 min while rotating at end-over-end at 1 rpm. After two washes with 75% ethanol, DNA was eluted in 50 μL of TE buffer. DNA quality (integrity and concentration) was assessed on the 4,200 Tapestation system (Agilent, Santa Clara, CA, USA) and with the Qubit broad-range DNA kit. To enrich for DNA >10 kb, size selection was done using the Circulomics Short Read Eliminator XS kit. We sequenced a total of four libraries, each prepared with 1 μg of size-selected HMW DNA, following the manufacturer’s guidelines for the Ligation Sequencing Kit SQK-LSK109 (Oxford Nanopore Technologies, Cambridge, UK). Sequencing was done on the MinION sequencing device with the Flow Cell model R9.4.1 (Oxford Nanopore Technologies, Cambridge, UK) and the ONT MinKNOW Software.

An Illumina sequencing library was prepared using a NebNext Ultra DNA II Kit (New England Biolabs, Ipswich, MA, USA) and was sequenced on a HiSeq X10 (150 bp paired end reads) by Novogene (Beijing, China).

### Mitogenome assembly, annotation and analysis

The Guppy base caller ONT v.3.2.4 was used for high-accuracy base calling on the raw sequence data, and only high-quality sequences with a Phred score >13 were used for the de novo mitogenome assembly with the program Flye v.2.5 (Kolmogorov et al., 2019) in the metagenome assembly mode. The method recovered the full circular assembly and to verify its accuracy, we first mapped the original reads back to the generated mitogenome assembly using Minimap2 (Li, 2018) with the following parameters: -k15—secondary = no -L -2. Second, we used BWA-MEM (Li, 2013) to map short-read Illumina sequences obtained from the whole-genome sequencing of another *O. rhinoceros* female collected from the same geographic location as the specimen used for the mitogenome assembly. The read alignment analysis in Pilon (Walker et al., 2014) was used to identify inconsistencies between the draft mitogenome assembly and the aligned short Illumina reads, removing small indels that represent homopolymers (e.g., >4 bp single nucleotide stretches) as an inherent sequencing error of the ONT (Mikheyev & Tin, 2014). Finally, we manually inspected if the Pilon correction occurred only in putative homopolymer regions by comparing the draft assembly with the Pilon-polished version.

The complete mitogenome sequence was initially annotated using the MITOS web server (Bernt et al., 2013), and tRNA genes and their secondary structures were cross-analysed using tRNAscan-SE v2.0 (Chan et al., 2019). To further refine the annotation and to examine mitogenome transcription, we used BWA-MEM to align Illumina reads from a transcriptome study of *O. rhinoceros* larvae (Shelomi, Lin & Liu, 2019), retrieved from the NCBI (SRR9208133). Finally, we manually inspected and compared our annotation to the complete and near complete mitogenome annotations of other related taxa (Table 1) in Geneious Prime (Biomatters development team, 2020). MEGA X (Kumar et al., 2018) was used to assess the codon usage and nucleotide composition of protein-coding genes. We used Geneious Prime to test if the nucleotide sequence of the *cox1* gene recovers the recently reported PCR-RFLP marker (Marshall et al., 2017). This was done by aligning the sequences of the primer pair (LCO1490 and HCO2198) to isolate the amplicon fragment and perform in silico restriction digestion with MseI restriction enzyme. The restriction digestion of the amplicon produces a set of fragment lengths that distinguishes CRB-G from other haplotypes (Marshall et al., 2017). The presence of tandem repeats within the control region was assessed with the Tandem Repeats Finder v.4.0.9 (Benson, 1999) using default parameters. The annotated mitogenome sequence has been deposited in GenBank under accession number MT457815.1.

**Table 1:** Taxa with complete or partial mitogenome sequences used for the phylogenetic analyses.

Accession	Reference	Organism	Genome type	Missing genes	Contains control region	Sequence length (bp)
FJ859903.1	Cameron et al. (2009)	*Rhopaea magnicornis*	Complete	None	Yes	17,522
JX412731.1	Timmermans et al. (2016)	*Cyphonistes vallatus*	Partial	*nd1*	No	11,629
JX412734.1	Timmermans et al. (2016)	*Trox* sp.	Partial	*nd2; cox1*	No	11,622
JX412739.1	Timmermans et al. (2016)	*Schizonycha* sp.	Partial	*nd2*	No	13,542
JX412755.1	Timmermans et al. (2016)	*Asthenopholis* sp.	Partial	*nd2*	No	12,352
KC775706.1	Kim et al. (2014)	*Protaetia brevitarsis*	Complete	None	Yes	20,319
KF544959.1	Kim & Kim (2013)	*Polyphylla laticollis mandshurica*	Partial	None	No	14,473
KU739455.1	Breeschoten et al. (2016)	*Eurysternus foedus*	Partial	None	No	15,366
KU739465.1	Breeschoten et al. (2016)	*Coprophanaeus* sp.	Partial	None	No	15,554
KU739469.1	Breeschoten et al. (2016)	*Bubas bubalus*	Partial	None	No	16,035
KU739498.1	Breeschoten et al. (2016)	*Onthophagus rhinolophus*	Partial	None	No	15,237
KX087316.1	A. Hunter, 2017 (unpublished)	*Melolontha hippocastani*	Partial	None	No	15,485
MN122896.1	A. Margaryan, 2019 (unpublished)	*Anoplotrupes stercorosus*	Partial	*nd2*	No	13,745
NC_030778.1	Kim et al. (2016)	*Osmoderma opicum*	Complete	None	Yes	15,341
NC_038115.1	Yang et al. (2018)	*Popillia japonica*	Complete	None	Yes	16,541

### Phylogenetic analysis

To ascertain if our newly sequenced *O. rhinoceros* mitogenome can be correctly placed within the Dynastinae subfamily of the Scarabaeidae family, we performed the phylogenetic analyses with 15 additional taxa for which complete or near complete mitogenome sequences could be retrieved from the NCBI. We used thirteen species from five subfamilies of the Scarabaeidae family (Dynastinae, Rutelinae, Cetoniine, Melolonthinae, Scarabaeinae) and members of two other families from Scarabaeoidea as outgroups (Trogidae, Geotrupidae) (Table 1).

Nucleotide sequences of all 13 protein-coding genes (PCGs) were first translated into amino acid sequences under the invertebrate mitochondrial genetic code and aligned using the multiple alignment in Geneious Prime. The aligned amino acid matrix was back-translated into the corresponding nucleotide matrix and the Perl script Degen v1.4 (Zwick, Regier & Zwickl, 2012; Regier et al., 2010) was used to create the degenerated protein-coding sequences in order to reduce the bias effect of synonymous mutations on the phylogenetic analysis. These final alignments from all 13 PCGs were concatenated using Geneious Prime.

We estimated the phylogeny using two methods: the Maximum likelihood (ML) inference implemented in IQ-TREE web server (Trifinopoulos et al., 2016) and the Bayesian inference (BI) in MrBayes (Huelsenbeck & Ronquist, 2001). For the ML analysis, the automatic and FreeRate heterogeneity options were set under optimal evolutionary models, and the branch support values were calculated using the ultrafast bootstrap (Hoang et al., 2018) and the SH-aLRT branch test approximation (Shimodaira & Hasegawa, 1999) with 1,000 replicates. The Akaike information criterion (AIC) in ModelFinder (Kalyaanamoorthy et al., 2017) was used to select the best substitution model, and the BI phylogeny was generated with a total chain length of 1,100,000 (burn-in of 110,000 trees) and sampling every 200 cycles. The consensus trees with branch support were viewed and edited in Figtree v1.4.2 (Rambaut, 2014).

## Results

### Mitogenome assembly, organization and transcription

The ONT long reads enabled the complete assembly of the circular mitochondrial genome for *O. rhinoceros* with just one MinION flow cell. This initial assembly was 20,954 bp in length and had a coverage of 3,834x. Based on the coverage ratio between the mitochondrial and draft nuclear genome assembly (unpublished data), we estimated that there were 320 copies of mitogenome for every nuclear genome copy. After including the data from three additional MinION flow cells, we produced a draft mitogenome assembly that was 21,039 bp long and had a coverage of 10,292x. Again, based on the coverage ratio between the mitochondrial and draft nuclear genome assembly, we estimated that there were 294 mitogenome copies per one copy of the nuclear genome in the total dataset. We then used the aligned Illumina reads to identify and correct indels, which are the most common error type produced by the ONT sequencing (Mikheyev & Tin, 2014), resulting in the removal of 141 bp. Of those, 95 bp were removed from the 5- to 9-mers (homopolymers). The final polished assembly was 20,898 bp in length. Our Illumina-only assembly, on the other hand, recovered a 17,665 bp mitogenome length with 3,233 bp missing from the control-region (CR).

The nucleotide composition of the complete mitogenome had high A + T bias (37.7% A, 32.8% T, 19.4% C and 10% G), and the long CR matched this genome-wide pattern (34.3% A, 35.8% T, 20.4% C and 9.5% G). The annotation revealed all 37 genes, with the rearrangement of *trnI* and *trnQ* genes, that showed the order: CR*-trnQ-trnI-trnM-nad2* instead of CR-*trnI-trnQ-trnM- nad2* (Table 2). CR resides within a large non-coding region (6,204 bp long) located between *rrnS* and *trnQ*, and the tandem repeats analysis revealed a complex structure of this large region with 11 putative repeats that had a consensus sequence between 7 and 410 bp repeated 2–12 times (Table 3). The length of 22 tRNAs ranged from 63 to 70 bp (Table 2), and their predicted secondary structures exhibited a typical clover-leaf structure. The length of *rrnL* and *rrnS* were 1,283 bp and 783 bp, respectively (Table 2).

**Table 2:** Organization of the newly sequenced mitogenome of *Oryctes rhinoceros*.

Feature name	Type	Start position	End position	Length	Direction	Start codon	Stop codon
*trnQ*	tRNA	1	69	69	Reverse		
*trnI*	tRNA	127	190	64	Forward		
*trnM*	tRNA	195	263	69	Forward		
*nad2*	gene	276	1,271	996	Forward	ATT	TAA
*trnW*	tRNA	1,270	1,335	66	Forward		
*trnC*	tRNA	1,328	1,392	65	Reverse		
*trnY*	tRNA	1,393	1,456	64	Reverse		
*cox1*	gene	1,458	2,993	1,536	Forward	ATC	TAA
*trnL2*	tRNA	2,989	3,054	66	Forward		
*cox2*	gene	3,055	3,762	708	Forward	ATA	TAA
*trnK*	tRNA	3,743	3,812	70	Forward		
*trnD*	tRNA	3,813	3,875	63	Forward		
*atp8*	gene	3,876	4,031	156	Forward	ATT	TAA
*atp6*	gene	4,025	4,696	672	Forward	ATG	TAT
*cox3*	gene	4,695	5,483	789	Forward	ATG	TAT
*trnG*	tRNA	5,482	5,545	64	Forward		
*nad3*	gene	5,546	5,899	354	Forward	ATC	TAG
*trnA*	tRNA	5,898	5,962	65	Forward		
*trnR*	tRNA	5,963	6,027	65	Forward		
*trnN*	tRNA	6,028	6,092	65	Forward		
*trnS1*	tRNA	6,093	6,159	67	Forward		
*trnE*	tRNA	6,161	6,224	64	Forward		
*trnF*	tRNA	6,223	6,288	66	Reverse		
*nad5*	gene	6,287	8,005	1,719	Reverse	ATT	TAT
*trnH*	tRNA	8,003	8,066	64	Reverse		
*nad4*	gene	8,066	9,403	1,338	Reverse	ATG	TAA
*nad4l*	gene	9,397	9,687	291	Reverse	ATG	TAA
*trnT*	tRNA	9,690	9,754	65	Forward		
*trnP*	tRNA	9,755	9,819	65	Reverse		
*nad6*	gene	9,821	10,321	501	Forward	ATC	TAA
*cob*	gene	10,321	11,463	1,143	Forward	ATG	TAG
*trnS2*	tRNA	11,462	11,527	66	Forward		
*nad1*	gene	11,547	12,497	951	Reverse	ATT	TAA
*trnL1*	tRNA	12,499	12,561	63	Reverse		
*rrnL*	rRNA	12,559	13,841	1,283	Reverse		
*trnV*	tRNA	13,843	13,912	70	Reverse		
*rrnS*	rRNA	13,912	14,694	783	Reverse		
Control region	misc_feature	14,695	20,898	6,204	None		
Expressed region 1	misc_RNA	15,117	15,253	137	Reverse		
Expressed region 2	misc_RNA	15,321	15,476	156	Reverse		
Expressed region 3	misc_RNA	17,744	18,387	644	Reverse		

**Table 3:** Characteristics of the putative tandem repeats in the control region of the *Oryctes rhinoceros* mitogenome.

Indices	Period Size	Copy Number	Consensus Size	Percent Matches	Percent Indels	Score	A	C	G	T	Entropy (0–2)
14,764–14,795	16	2	16	93	0	55	53	0	0	46	1
14,757–14,797	7	6	7	78	16	50	48	0	0	51	1
14,818–14,859	15	3	14	86	10	59	38	0	0	61	0.96
15,012–15,440	133	3.2	133	97	1	824	52	15	6	25	1.65
15,447–17,166	285	6	285	98	0	3,336	24	29	13	33	1.93
16,955–17,949	206	4.9	205	98	1	1,933	24	29	12	33	1.92
16,955–17,949	410	2.4	409	98	1	1,938	24	29	12	33	1.92
18,024–18,419	110	3.6	110	96	1	724	41	24	8	25	1.83
18,656–18,707	22	2.3	23	81	15	65	51	1	3	42	1.31
19,664–20,895	102	12	102	98	0	2,351	36	11	7	44	1.69
19,664–20,895	205	6	204	98	0	2,369	36	11	7	44	1.69

All protein coding genes (PCGs) started with a standard initiation codon (ATN), 10 of 13 PCGs terminated with the conventional stop codons (TAG or TAA), while three genes (*atp6*, *cox3* and *nad5*) had an incomplete stop codon T (Table 2). In silico digestion of the *cox1* amplicon (delineated with the primer sequences from (Folmer et al., 1994)) produced the fragments 253 bp-, 138 bp-and 92 bp-long (Fig. S1) that are diagnostic for the CRB-G haplotype (Marshall et al., 2017).

**Figure 1:**
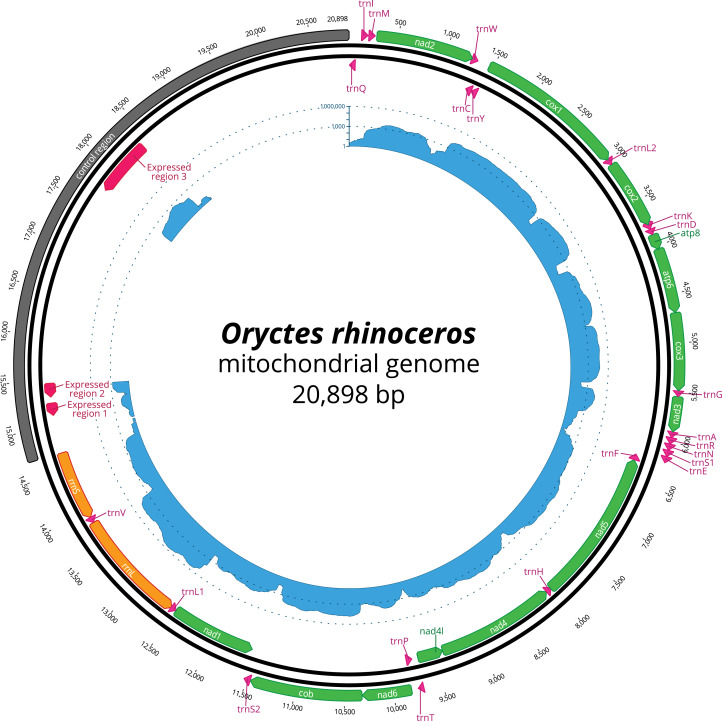
Circular representation of complete *O. rhinoceros* mitochondrial genome. The position and orientation of 13 PCG genes (green), 22 trna genes (pink), two rRNA genes (orange), control region (grey) with three expressed domains (red). The inner circle displays transcriptome read depth (blue) on a logarithmic scale.

The mapping of the transcriptome sequencing reads to the newly assembled mitogenome revealed that all PCGs were transcribed (mean coverage depth per base >23000X, Fig. 1), with *cox1* and *cox2* showing the highest level of expression when compared to the rRNA genes in the examined larval samples (Fig. 1). We found three domains within the large CR-containing region that also showed detectable transcription levels, with the transcript sizes of 137, 156 and 644 bp respectively. Our attempts to annotate these transcripts were not successful due to the fact that their sequences did not contain any open reading frames, nor did they have any significant BLAST hits within the NCBI’s reference RNAseq or nucleotide databases.

### Phylogenetic analysis

The alignment of concatenated PCG sequences of 16 species (degenerated by Degen script (Zwick, 2013)) had 1,449 parsimony-informative sites, 1,818 singleton sites and 7,823 constant sites. ModelFinder identified GTR+F+R3 as the optimal substitution model (AIC: 94067.741, AICc: 94068.053), and given the proportion of invariable sites of 0.469 and the estimated gamma shape alpha of 0.728, we used the GTR+I+G model as the available alternative in MrBayes. This is the most parameter-rich model that has been shown to yield highly robust tree topologies for long alignments (>1,250 bp) in the smaller sample sizes (<17 taxa), irrespective of the model selection results (Abadi et al., 2019).

**Figure 2:**
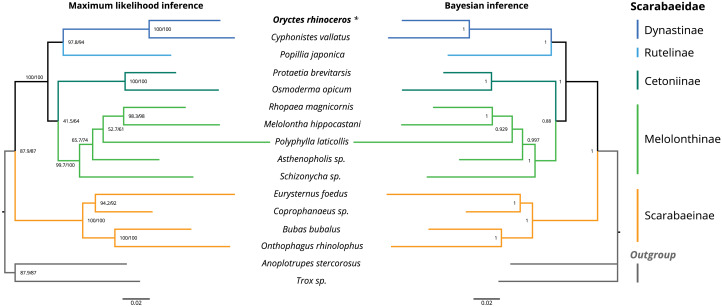
Maximum likelihood (ML) and Bayesian (BI) consensus tree inferred from the PCG dataset using IQ-TREE (ML) and MrBayes (BI). Branch support values for the ML consensus tree are presented near each node as SH-aLRT support (%)/ultrafast bootstrap support (%), and branch lengths were optimized by maximum likelihood on original alignment. Branch support values for the BI consensus tree are posterior probabilities (0–1).The colored lines correspond to Scarabaeidae subfamilies.

The topology of the maximum likelihood (IQ-tree) phylogeny was identical to the Bayesian (MrBayes) phylogeny, with *O. rhinoceros* being grouped with another member of the Dynastinae subfamily (*Cyphonistes vallatus*) with high confidence (100% SH-aLRT support, 100% ultrafast bootstrap support in ML, posterior probability 100% in BI). Dynastinae and Rutelinae were inferred as sister clades, that together with Cetoniine and Melolonthinae formed a basal split between phytophagus and coprophagus scarab beetles (Scarabaeinae) (Fig. 2).

## Discussion

The complete circular mitogenome sequence of *O. rhinoceros* is the only complete mitogenome assembly and annotation for the Dynastinae subfamily, and among only a few complete mitogenomes for the scarab beetles. Its size (20,898 bp) is among the largest reported in Coleoptera, and is similar in size to another scarab beetle, *Protaetia brevitarsis* (20,319 bp) (Kim et al., 2014). Mitogenome size is driven by the large non-coding control region (CR) that is 6,204 bp-and 5,654 bp-long in *O. rhinoceros and P. brevitarsis*, respectively. In *Popillia mutans*, another scarab beetle with a complete mitogenome sequence, the reported length of this region is only 1,497 bp (Song & Zhang, 2018).

Variation in the size and nucleotide composition of the mitochondrial CR is not unusual in insects (Zhang & Hewitt, 1997), however, there could also be technical reasons for some size discrepancies among taxa. Given that we recovered 17,665 bp in our Illumina-only assembly (3,233 bp were missing from the CR), it is possible that many other beetles have larger mitogenomes, but the repetitive content of CR presents a challenge for the most commonly used approaches such as Sanger or short-read NGS sequencing of long-PCR amplicons (Cameron, 2014a). While the CR size can be estimated from the size of long-PCR amplicons, its Sanger sequencing by primer walking is often impossible because it is difficult to design useful primers where GC content is insufficient and homopolymers and tandem repeats are abundant (Cameron et al., 2012; Cameron, 2014a). Long repetitive regions also complicate the assembly process with the short-read NGS sequences (either from the long-PCR amplicons or total DNA extractions) because they are often algorithmically collapsed due to their similarity. For example, three out of five scarab mitogenome assemblies recently generated with the short-read technology are incomplete and lack the control region and adjacent genes (Song & Zhang, 2018). The use of RNA-seq transcriptome data is the least likely to recover the CR, given that this part of the mitogenome generally does not transcribe (or has few short domains of transcription, as we describe in *O. rhinoceros*).

Our approach included the long-read (ONT) sequencing of the libraries that were prepared with non-PCR-amplified DNA, resulting in a fully closed circular assembly with thousands of reads spanning the entire length of the control region. The superiority of long-read sequencing technologies to capture the long repeated, AT-rich sequences has led to the discovery of remarkable interspecific variation in the length of the intergenic repeat regions in the mitogenomes of seed beetles (Chrysomelidae), that can range between 0.1 and 10.5 kbp (Sayadi et al., 2017). However, for taxa where CR is short (e.g., Lepidoptera (Zhang & Hewitt, 1997)), the long-read technology is not necessary for obtaining the complete mitogenome assemblies.

Unambiguous detection of gene rearrangements and other structural changes is another benefit of the long-read sequencing. In the *O. rhinoceros* mitogenome, *trnQ* gene precedes *trnI* gene, and this rearrangement is supported with thousands of long reads spanning this region. The rearranged position of *trnI* and *trnQ* genes is found in almost all species of Hymenoptera (Dowton et al., 2009), and was also reported in flatbugs (Hemiptera, Aradidae) (Song et al., 2016). A number of other rearrangements in tRNA genes have been reported in Lepidoptera and Neuroptera (Cao et al., 2012; Cameron et al., 2009), and because they all occurred between the CR and *cox1*, it has been hypothesized that this might be a ‘hotspot’ region for such changes (Dowton et al., 2009).

It is also important to note the higher basecalling error rate of ONT when compared to the short-read NGS or Sanger data. Using the aligned short-read sequences (Illumina, San Diego, CA, USA), we identified 141 erroneous additions of 1 or 2 nucleotides, and 67% of those were found in 5-to 9-bp homopolymers. This occurred despite achieving the depth of thousands of reads per each nucleotide position (>10k × median depth), indicating systematic errors during the basecalling process that result in erroneous consensus sequence (Wick, Judd & Holt, 2019), and this cannot be circumvented with high sequencing depth alone. When short-read data are not available for polishing, we suggest to carefully manually inspect the alignment between the draft genome and other high-quality assemblies from the related taxa in order to remove most (if not all) errors in the consensus sequence.

The nucleotide composition of the final (polished) *O. rhinoceros* mitogenome sequence had high A + T bias (37.7% A, 32.8% T, 19.4% C and 10% G), which is highly concordant with other scarab beetle species (Song & Zhang, 2018), and the long CR matched this genome-wide pattern (34.3% A, 35.8% T, 20.4% C and 9.5% G). All PCGs start with a standard ATN codon, including *cox1* that generally seems to be a hot-spot for non-canonical start codon in annotations of invertebrate genomes (Donath et al., 2019). Among 15 other Scarabaeoidea we examined (Table 1), 14 had *cox1* in their mitogenome assembly, nine of which had a standard ATN codon while 5 taxa did not have a defined start codon, resulting in a different length of this gene. A more comprehensive analysis (with an extended taxon sampling) is needed to reliably quantify the extent of *cox1* start-codon variation in this insect group. We also found evidence of some transcriptional activity within the control region of the *O. rhinoceros* mitogenome, but to fully characterize this pattern, more transcriptome data (from different tissues, life stages etc.) would need to be tested. Transcriptional activity within the intergenic repeat regions has been detected in mitogenomes of seed beetles (Sayadi et al., 2017), suggesting that ‘mitochondrial dark matter’ could be a source of non-coding RNAs in insects.

We used phylogenetic analysis to assess the quality of the *O. rhinoceros* mitogenome assembly, expecting to recover its grouping with one other member of the Dynastinae subfamily. This grouping was indeed highly supported in both ML and BI phylogeny (Fig. 2). Identical topology of the ML and BI trees showed Dynastinae and Rutelinae as sister clades, that formed a basal split between phytophagous and coprophagous scarab beetles with Cetoniine and Melolonthinae (Fig. 2). It is worth noting that the intra-family phylogeny of this group is inconsistent depending on the type of the sequence data (mitogenome vs. mito+nuclear genes) used for the inference. While Melolonthinae have been reported as paraphyletic and Cetoniinae as more closely related to Dynastinae and Rutelinae, the sister-group relationship between Dynastinae and Rutelinae was recovered in all previous studies of this group (Ahrens, Schwarzer & Vogler, 2014; Gunter et al., 2016; Song & Zhang, 2018). Further support for the high quality of the *O. rhinoceros* mitogenome sequence and annotation was our in silico recovery of the correct PCR-RFLP marker set that is diagnostic for the invasive CRB-G haplotype (Marshall et al., 2017).

## Conclusions

We report the circularized complete mitochondrial genome assembly and annotation for *O. rhinoceros*, the major insect pest of coconut and oil palms. The long-read ONT sequencing allowed us to identify structural variation (*trnI-trnQ* rearrangement) and span the assembly across the entire 6,203 bp-long control region that contains tandem repeats and regions of transcriptional activity. This high-quality genomic resource facilitates future development of a molecular marker toolset to help with the biosecurity and management efforts against this resurgent pest. As the first complete mitogenome for the genus *Oryctes* and the subfamily Dynastinae, and among a few for the entire scarab beetle family (Scarabaeidae), it will contribute to the resolution of higher-level taxonomy and phylogeny of phytophagous scarab beetles that remain understudied despite containing many agricultural pests.

## Supplemental Information

10.7717/peerj.10552/supp-1 Supplemental Information 1 Graphical representation of in silico PCR-RFLP marker analysis. The amplicon from the partial *cox1* gene is delineated with forward and reverse universal *cox1* primers (Folmer et al., 1994) and MseI restriction enzyme recognition sites are marked. The diagnostic SNP (Marshall et al., 2017) A>G is marked in orange. This analysis generates in silico fragments 253bp, 138bp, 92bp, 28bp and 13bp-long. The fragments 253, 138 and 92bp are visible on a 2% agarose gel in (Marshall et al., 2017) and are diagnostic for the CRB-G haplotype.

## Additional Information and Declarations

### Field Study Permissions

The following information was supplied relating to field study approvals (i.e., approving body and any reference numbers): Mrs Helen Tsatsia (Director of Research) and members of the research team at the Ministry of Agriculture and Livestock, Honiara, Solomon Islands Government facilitated the insect collection in Solomon Islands.

### Data Availability

The following information was supplied regarding data availability: The assembled data is available at NCBI: MT457815.1.
