# Supplementary figures and images for: The complete mitochondrial genome sequence of Oryctes rhinoceros (Coleoptera: Scarabaeidae) based on long-read nanopore sequencing
Source: PeerJ. 2021 Jan 13;9:e10552. doi: 10.7717/peerj.10552 (PMC7811291; doi:10.7717/peerj.10552)

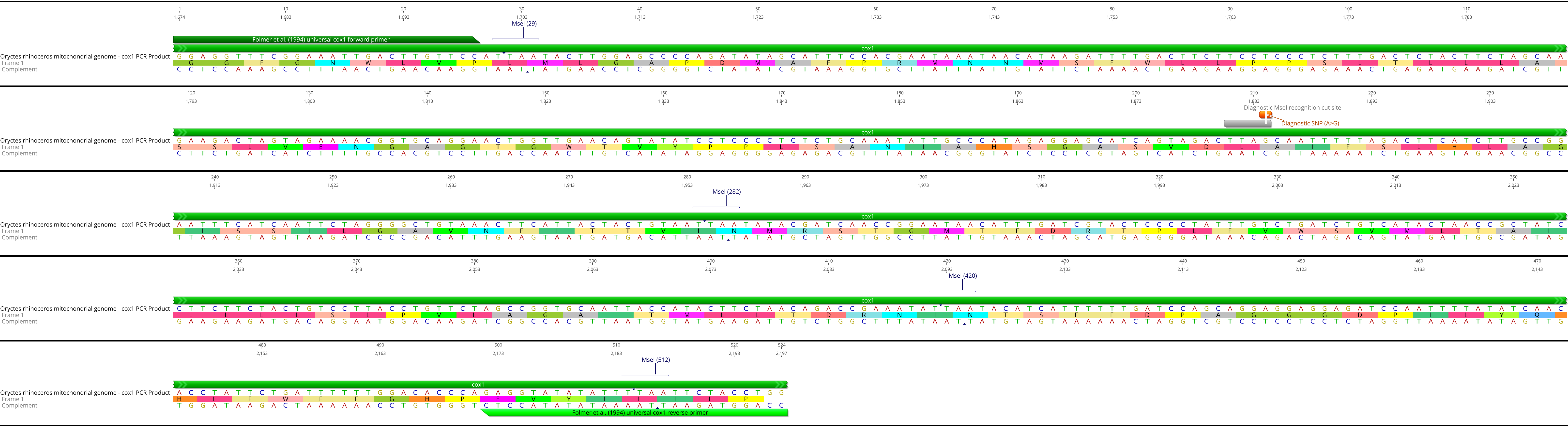

Supplement: Supplemental Information 1 — The amplicon from the partial cox1 gene is delineated with forward and reverse universal cox1 primers (Folmer et al., 1994) and MseI restriction enzyme recognition sites are marked. The diagnostic SNP (Marshall et al., 2017) A>G is marked in orange. This analysis generates in silico fragments 253bp, 138bp, 92bp, 28bp and 13bp-long. The fragments 253, 138 and 92bp are visible on a 2% agarose gel in (Marshall et al., 2017) and are diagnostic for the CRB-G haplotype. [file peerj-09-10552-s001.pdf]
